# The Influence of Farming Systems, Genotype and Their Interaction on Bioactive Compound, Protein and Starch Content of Bread and Spelt Wheat

**DOI:** 10.3390/foods11244028

**Published:** 2022-12-13

**Authors:** Verica Takač, Viola Tóth, Marianna Rakszegi, Péter Mikó, Sanja Mikić, Milan Mirosavljević

**Affiliations:** 1 Institute of Field and Vegetable Crops, Maksima Gorkog, 21000 Novi Sad, Serbia; 2 Agricultural Institute, Centre for Agricultural Research, Brunszvik u. 2, 2462 Martonvásár, Hungary

**Keywords:** spelt, organic farming, bioactive compound, alkylresorcinol, fructan, arabinoxylan

## Abstract

An increase in the production and consumption of spelt products can be associated with positive effects on human health, which are attributed to bioactive compounds present in the grain. The basic success of spelt wheat in organic farming might be explained by the fact that spelt wheat belongs to the group of hulled wheat where the presence of a husk protects the seed from abiotic and biotic stress factors, thus demanding less chemical protection. The goal of this study was to investigate the variations in the bioactive compound (alkylresorcinol, arabinoxylan, β-glucan), protein, starch and fructan content of bread and spelt wheat under different farming systems (conventional and organic). The results showed higher protein and alkylresorcinol but lower fructan content in spelt wheat. Organic spelt had significantly higher starch, fiber and alkylresorcinol content but lower β-glucan and protein content than conventionally grown spelt. The spelt variety ‘Oberkulmer-Rotkorn’ was characterized by the highest values for the majority of analyzed traits under both farming systems. Overall, the environmental conditions (Hungary and Serbia), farming systems (conventional and organic) and wheat species (bread and spelt) contributed to the variations of the compositional traits in different manners.

## 1. Introduction

As one of the big three cereals widely grown for their abundance of nutrients, wheat (*Triticum* sp.) has been studied in many aspects and by many researchers. Besides its dominant role in supplying the food industry with white bread flour, nowadays, there is an increasing range of food products present in the market containing wholemeal flour as a prevalent source of carbohydrates. This increase in the production and consumption of wholemeal wheat products can be associated with the positive effects of wholemeal flour and its main constituents on human health [1]. These positive effects are attributed to the presence of important nutrients in higher quantities, including essential amino acids, minerals and vitamins and, especially, dietary fibers. Wheat is a major source of dietary fiber in the human diet [2]. The most important of all positive effects of dietary fiber is associated with its ability to act as a prebiotic [3] as it serves as a carbon and energy source for the growth of bifidobacteria [4], the beneficial bacteria in the gut. Although these carbohydrates are highly indigestible in the upper gastrointestinal tract, they are fermented by beneficial bacteria in the colon [5]. In this way, dietary fiber promotes the development of beneficial bacteria, which generate short-chain fatty acids supplying the host with energy and lowering the pH value in the bowel [3].

Milling of wheat grain isolates the starchy endosperm. The other two parts of wheat grain—the germ, and the bran with aleurone layer—are removed. Despite the fact that those two fractions represent about 2.5–3% and 10–14% by weight, respectively [6], they contain much more bioactive components with high nutritional value than the biggest fraction of wheat grain, the starchy endosperm (representing 80–85% by weight). While the endosperm is mainly composed of carbohydrates, starch and sugars, the germ is rich in proteins, lipids and sugars. The third grain fraction, the bran, contains practically all the bioactive compounds, with dietary fiber being the most abundant [7]. These non-starch polysaccharides, which include soluble and insoluble dietary fibers, comprise arabinoxylan, cellulose, lignin, fructan and β-glucan. The largest portion of bran dietary fibers belongs to soluble arabinoxylan, followed by smaller amounts of cellulose, lignin, fructan and in the lowest amount β-glucan [7]. Approximately half of the total dietary fiber of the whole wheat grain belongs to arabinoxylan, the dominant cell-wall polysaccharide [8]. Dietary fiber includes mixtures of various non-starch polysaccharides, which together account for 90% of the dietary fiber content in wheat and 45–55% of dietary fiber in bran. On the other side, the dietary fiber in the endosperm is represented in less than 3%. Other bioactive compounds present in the bran include minerals, vitamin B complex and some phytochemical compounds, especially antioxidants such as phenolic compounds [7]. Alkylresorcinol is one of the phytochemical compounds present only in the bran; more precisely, in the outer layer of the seed coat, the testa [9]. As it can be found only in the bran fraction, it can be used as a biochemical marker of wholemeal wheat flour and bran in food [10]. Its relative quantity in the bran exceeds the relative amount in whole grain by four times [7]. Generally, the synergistic activity of bioactive compounds in the bran and germ has a protective role in humans, contributing to health. Despite the fact that they represent less than 15% of the wheat grain, the role of the wheat bran fraction is multifunctional. Besides protecting the grain from microorganisms, insects and weather conditions, another important function of wheat bran—and, particularly, bioactive compounds present in it—is its role in protecting the organism against many chronic diseases, including metabolic syndrome [11], type 2 diabetes, obesity, cardiovascular diseases [12], respiratory system diseases [13] and cancer [14]. According to Stevenson et al. [15], the health benefits of wheat bran arise from nutrients (nutritional effects), dietary fibers (mechanical effects on digestive system) and phytonutrients, such as phenolic acid and alkylresorcinol (antioxidant effects).

Conventional agriculture relies on the chemical control of pathogens and weeds by applying pesticides and synthetic fertilizers during the vegetation period [16] in order to gain higher and more stable yields. Significant use of chemicals, such as the application of synthetic chemicals in conventional farming, leads to the buildup of heavy metals, especially cadmium, in the soil [17]. According to the Food and Agriculture Organization of the United Nations (FAO), organic agriculture is defined as holistic production that improves the biodiversity, biological cycles and activity of soil organisms, fostering agroecosystem well-being [18]. The main principles used in an organic farming system rely on the usage of non-synthetic pesticides and fertilizers from organic sources. As a result, the physical and chemical qualities of soil along with its benefits for insects and other micro- and macrofauna are improved [19], pollution is reduced and, thus, the preservation and protection of the environment is achieved [20], which is ultimately beneficial to human health. Although organic farming is more energy efficient than conventional methods [21], the main disadvantage of organic production is lower yield [19], the effect of which is reflected in product prices [22]. An advantage of spelt wheat in organic farming might be the presence of husk that protects the seed from abiotic and biotic stress factors [23]. Furthermore, spelt plants have better weed suppression abilities than bread wheat plants, which help to reduce or avoid the use of herbicides [24].

The bioactive component composition of spelt wheat has been investigated and compared to other cereal species [25,26]. However, their findings are sometimes contradictory, probably because most of these studies include few genotypes. Some general observations have been made so far on the advantages of spelt’s compositional traits, such as the higher protein content compared to wheat [27], but the results of other components are rather inconsistent. Thus, the aim of our study was (1) to investigate differences in the bioactive compound (arabinoxylan, β-glucan, alkylresorcinol, fructan), protein and starch content, as well as quality traits, between bread and spelt wheat; (2) to determine the influence of organic and conventional field management systems on two different hexaploid wheat species, under two different field management systems (organic, conventional) in Hungary and Serbia; and (3) to find the best varieties suitable for cultivation under specific management systems.

## 2. Materials and Methods

### 2.1. Plant Material and Field Experiment

Five bread and five spelt wheat varieties from five European countries (Table 1) were grown during two growing seasons (2018/2019 and 2019/2020) under a conventional production field trial of the Institute of Field and Vegetable Crops, Novi Sad in Serbia (conversion to organic agriculture has not been implemented) and during the three consecutive growing seasons (2018/2019, 2019/2020 and 2020/2021) under conventional and organic systems at the experimental field of the Center for Agricultural Research, Martonvásár, in Hungary.

At the field trial of the Institute of Field and Vegetable Crops in Serbia, the varieties were sown in 5 m^2^ plots in a randomized complete block design with three replications on chernozem-type soil with soybean as a previous crop (Table 2). In 2018, prior to sowing, ca. 50 kg N/ha, 60 kg P/ha and 60 kg K/ha of fertilizers were applied with an additional top-dressing of 50 kg N/ha of ammonium nitrate (33% N) at the beginning of February, based on the N-min analysis. Spring weeds were controlled with herbicides (25 g/ha Stockstar, Stockton Chemical, Stockton, CA, USA, containing 500 g/ha tribenuron-methyl and 0.6 L/ha Lodin EC, HELM AG, Hamburg, Germany, containing 360 g/L fluroxypyr). Pests were managed with 50 mL/ha Vantex CS (FMC Corporation, Philadelphia, PA, USA) containing 60 g/L gama-cyhalothrin. In May, 1 L/ha of the fungicide Prosaro 250 EC (Bayer CropScience, Monheim am Rhein, North Rhine-Westphalia, Germany) containing 125 g/L tebuconazole + 125 g/L prothioconazole) was applied. In 2019, the same fertilizer was applied before sowing, and a further 50 kg N/ha of ammonium nitrate (33% N) was top-dressed as according to N-min analysis. Treatment with the herbicide Biathlon 4D (BASF SE, Ludwigshafen, Germany), containing 714 g/kg tritosulfuron and 54 g/kg florasulam, and the fungicide Osiris (2 L/ha, BASF SE, Ludwigshafen, Germany), containing 37.5 g/L epoxiconazole and 27.5 g/L metconazole, was applied in early April. In May 2019, the same treatment with Osiris was repeated in combination with the insecticide Decis 2.5 EC (0.3 L/ha, Bayer CropScience, Monheim am Rhein, North Rhine-Westphalia, Germany) containing 25 g/L deltamethrin. Weeds were removed by hand when required. Meteorological conditions in Serbia for two growing seasons are given in Table 3.

In Hungary, at the Center for Agricultural Research, Martonvásár, the plots were 4 × 1.2 m with 6 rows spaced 20 cm apart. The varieties were sown in two replicates on clayey chernozem with pH of 7.25, 2.8% *w*/*w* humus, 210 mg/kg P_2_O_5_ and 210 mg/kg K_2_O. The previous crops were facelia, oil radish and oil radish at the conventional location for the three prior years, respectively, while it was facelia, buckwheat and buckwheat, respectively (2018/2019, 2019/2020, 2020/2021), at the organic site. At the conventional site, 120 kg/ha of N was applied through NPK fertilizer. Herbicides (4 L/ha U-46 D-fluid SL, 500 g/L 2-methyl-4-chlorophenoxyacetic acid; 40 g/ha Granstar 50 SX, 50% tribenuron methyl) and insecticide (0.2 L/ha Karate Zeon 5CS, contains 50 g/L λ-cyhalothrin) were used twice a year. Fungicides were not used. No fertilizers or any chemicals were applied at the organic sites. Weeds were removed physically.

The cumulative precipitation was low in the 2019/2020 and 2020/2021 seasons in Hungary, especially in the last 100 days of the growing period. The mean temperature was the highest in 2019/2020 (9.48 °C), while, in Serbia, higher mean temperatures were observed for two growing seasons (11.5 °C and 11.6 °C, for 2018/2019 and 2019/2020, respectively). The absolute minimum temperature over a full season was the lowest in 2018/2019; however, there were cold days in the last 100 days of crop development in 2019/2020 and 2020/2021, with negative 3.6 °C and 6.5 °C in Hungary, respectively, and negative 5.4 °C absolute minimum temperatures in Serbia in the 2019/2020 growing season. The absolute maximum temperature was higher in Hungary in 2018/2019 (36 °C) and in 2020/2021 (37.4 °C), and so the number of hot days warmer than 30 °C was the lowest in 2019/2020.

Spelt and bread wheat had to go through collecting, threshing (Wintersteiger LD350, Wintersteiger AB, Arnstadt, Germany) and cleaning (Hal-drup DC-20, Haldrup GmbH, Ilshofen, Germany) before milling and laboratory analysis.

### 2.2. Analysis of the Quality Traits and Bioactive Compound, Protein and Starch Content

#### 2.2.1. Physical and Quality Traits of Bread and Spelt Wheat Varieties

Marvin System (MarviTech GmbH, Wittenburg, Germany) and FOSS Tecator 1241 (FOSS, Hilleroed, Denmark) instruments were used for determination of thousand kernel weight (TKW) and test weight, respectively, for the harvested grains according to the standard MSZ 6367/4-86 method [28]. A Perten Laboratory Mill 3100 instrument was used for the production of wholemeal samples, while a Chopin CD1 Laboratory Mill (CHOPIN Technologies, Villeneuve-la-Garenne, France) was used for the white flour, after conditioning the seeds to a moisture content of 15.5%. Water absorption, dough development time, dough stability, dough softening and quality number, as the most important dough properties, were determined by the Brabender Farinograph instrument (Brabender, Duisburg, North Rhine-Westphalia, Germany) according to the standard ICC 115/1 method [29]. The wet gluten content and gluten index (GI) were analyzed with a Glutomatic 2200 (Perten, Hamburg, Germany) instrument according to the standard ICC 137/1 [30] and ICC 155 methods [31]. A SediCom System [32] was used for the measurement of the Zeleny sedimentation according to the standard ICC 116/1 method [33]. The proteolytic activity of samples represented by the spread of gluten was measured by changes in gluten ball diameter over 1 h at room temperature.

#### 2.2.2. Bioactive Compound Content Analysis of Bread and Spelt Wheat Varieties

Total and water-extractable pentosans, of which arabinoxylan is the major component, were analyzed with a colorimetric method according to Douglas [34] in three replicates. The amount of mixed-linkage β-glucan in wholemeal samples was determined using a Megazyme kit (Megazyme, Bray, Ireland) according to the AACC 32–23.01 standard method [35]. Each sample was analyzed in two replications. The alkylresorcinols were extracted from whole grain by acetone and colorimetrically determined in duplicate by Fast Blue B salt according to the methods of Tłuścik et al. [36]. Fructan content was examined by Megazyme fructan HK assay kit (Megazyme, Ireland) according to the AACC 32.32.01 method [37].

#### 2.2.3. Protein and Starch Content Analysis of Bread and Spelt Wheat Varieties

Total protein content of wholemeal samples was measured by the Dumas method with an Elementar Rapid N III Analyzer (Elementar Analysensysteme GmbH, Langenselbold, Germany) according to the ICC 167 standard method [38]. The starch content of the harvested grains was evaluated by the NIR method with a FOSS Tecator 1241 (FOSS, Hilleroed, Denmark) instrument in agreement with the ICC 202 standard method [39]. Both analyses were measured in two replications. A Rapid Visco Analyser (RVA-3D, Newport Scientific Pty. Ltd., Warriewood, NSW, Australia) was used to study the starch pasting properties of wheat flour. A 20 min pasting profile was used, consisting of a 2 min hold at 50 °C, a 6 min heating period, a 4 min hold at 95 °C, a 4 min cooling period to 50 °C and a 4 min hold at 50 °C as according to the AACC 76-21 method [40] and Batey et al. [41]. A Chopin SDmatic instrument (Chopin Technologies, Villeneuve-la-Garenne, France) was used for the determination of starch damage in accordance with the standard ICC 172 method [42]. The analysis was performed in one replicate. The details of the average values of the bioactive compound, protein and starch content of three environments, two farming systems (organic, conventional) and two species are given in Table S1.

### 2.3. Statistical Analysis

Descriptive statistics for bioactive compound, protein and starch content from bread and spelt wheat grown using conventional and organic farming systems were presented by violin plots drawn by R software (R version 4.2.1). Analysis of variance (ANOVA) and Tukey’s test using SPSS Statistics 27.0 software (SPSS Inc., Chicago, IL, USA) was used to test differences among the mean values of wheat species, varieties, growing seasons and farming systems. Correlations between the analyzed bioactive compound, protein and starch content for wheat species were determined with Pearson’s correlation coefficient by R software. Principal component analysis (PCA) carried out in Statistica 6.0 (TIBCO Software Inc., Palo Alto, CA, USA) was used to study variation in the compositional as well as protein- and starch-related traits of the grain and flour and examine relationships between varieties and obtain clusters.

## 3. Results

### 3.1. Differences in Bioactive Compound, Protein and Starch Content between Bread and Spelt Wheat, Conventional and Organic Farming Systems and Serbia and Hungary

**Figure 1:**
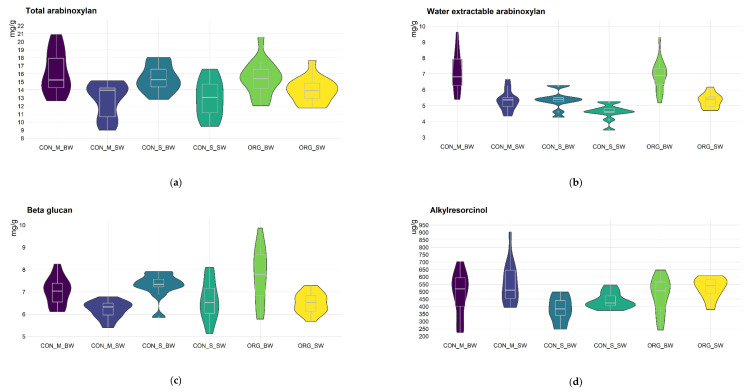

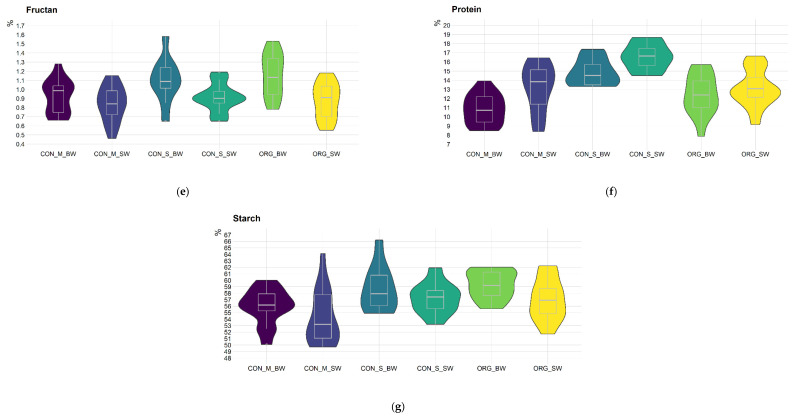
Violin plots for (**a**) Total arabinoxylan, (**b**) Water-extractable arabinoxylan, (**c**) β-glucan, (**d**) Alkylresorcinol, (**e**) Fructan, (**f**) Protein and (**g**) Starch content; CON—conventional farming system; M—Martonvásár, Hungary; S—conventional farming system in Serbia; ORG—organic farming system in Hungary; BW—bread wheat; SW—spelt wheat; minimum value—shown by the line below the violin plot; the first lower quartile (25th percentile)—shown by the lower end of the violin plot; median—shown by the horizontal line inside the plot; the third upper quartile (75th percentile)—shown by the upper end of the plot; maximum value—shown by the line above the violin plot.

Bioactive compound, protein and starch content showed variations between the two wheat species, two farming systems and two countries. The distribution for each group is shown with violin plots (Figure 1).

Total arabinoxylan content showed higher values in bread than in spelt wheat using conventional and organic farming systems. In bread wheat, total arabinoxylan content ranged from 12.0 to 20.9 mg/g with an average value of 15.7 mg/g, while in spelt wheat, it ranged from 9.0 to 17.7 mg/g with an average value of 13.3 mg/g. The highest value for this trait in bread wheat was found using the conventional farming system in Hungary (20.9 mg/g), while the lowest value was found using organic system in Hungary (12 mg/g). On the contrary, in spelt wheat, the lowest value (9 mg/g) was found with the conventional system in Hungary, whilst the highest value was found with the organic farming system (17.7 mg/g) in Hungary (Figure 1a).

Similar to total arabinoxylan content, the water-extractable arabinoxylan showed higher values in bread (6.58 mg/g) than in spelt wheat (5.13 mg/g) in different farming systems and countries (Figure 1b). Much higher values for water-extractable arabinoxylan in bread wheat were found in Hungary under conventional (7.1 mg/g) and organic (6.9 mg/g) farming systems than in Serbia under conventional production (5.4 mg/g). In spelt wheat, the average values were the same for conventional and organic farming in Hungary (5.3 mg/g), but in Serbia, the average value was lower (4.6 mg/g).

β-glucan content ranged from 5.8 to 9.9 mg/g and 5.1 to 8.1 mg/g in bread and spelt wheat, respectively (Figure 1c). Notably higher β-glucan content was seen in bread (7.4 mg/g) than in spelt (6.4 mg/g) wheat. In organic farming, bread wheat had a higher average (7.7 mg/g) than conventional farming in both countries. The bread wheat varieties grown using the conventional farming system in Serbia had a higher average value (7.3 mg/g) than in Hungary (7.0 mg/g). In spelt wheat, the highest average value (6.6 mg/g) was found at the conventional farming system in Serbia, followed by 6.4 mg/g at the organic farming system and 6.2 mg/g at the conventional farming system in Hungary.

Contrary to arabinoxylan and β-glucan content, the antioxidant capacity represented by alkylresorcinol content was remarkably higher in spelt wheat varieties (511.6 µg/g) than in bread wheat varieties (454.9 µg/g) in organically and conventionally grown farming systems (Figure 1d). The highest (702.4 µg/g) and the lowest (223 µg/g) alkylresorcinol content in bread wheat was found in samples originating from the conventional farming system in Hungary. In spelt wheat, alkylresorcinol content ranged from 372.6 µg/g in samples from Serbia to 902.4 µg/g in samples originating from the conventional farming system in Hungary. In both species, the lowest average value was found at the conventional farming system in Serbia (381.5 and 439.9 µg/g for bread and spelt wheat, respectively) followed by organic farming (477.0 and 525.1 µg/g for bread and spelt wheat, respectively). The highest average value, 483.6 µg/g for bread and 553.3 µg/g for spelt wheat, originated from the conventional farming system in Hungary (Figure 1d).

Bread wheat varieties were characterized by a higher fructan content (1.1%) than spelt wheat (0.9%), and it ranged from 0.7% to 1.6% in bread and from 0.5% to 1.2% in spelt wheat (Figure 1e). The highest values in bread wheat were found at the organic farming system in Hungary (1.53%) and conventional farming system in Serbia (1.58%), while the lowest values were detected at the conventional farming system in Hungary (0.66%) and Serbia (0.65%). Organically (1.18%) and conventionally grown spelt wheat varieties from Serbia (1.19%) were characterized by the highest fructan content. The conventional farming system in Serbia and the organic farming system in Hungary were characterized by the highest value for fructan content (0.9%), followed by the conventional farming system in Hungary (0.8%).

Differences were found in protein content among spelt and bread wheat as well as among farming systems (Figure 1f). Bread wheat varieties had lower protein content (12.11%) than spelt wheat varieties (14.33%). Protein content in bread wheat ranged from 7.85% to 17.4%, while the range was from 8.48% to 18.66% in spelt. The Serbian conventional farming system showed the highest protein content values for both species. On the other side, the lowest values were found for both spelt and bread wheat at the organic farming site in Hungary. Conventionally grown spelt wheat from Hungary and Serbia did not differ in protein content (15%), while the same organically grown varieties had lower values (13.1%). Similarly, conventionally grown bread wheat from Hungary and Serbia also had higher values for protein content (13.8% and 11.9%, respectively) than the organically grown ones (10.5%) (Figure 1f).

Bread wheat generally had higher starch content (58.4%) than spelt (55.4%) (Figure 1g). The starch content in bread wheat was higher at the organic site in Hungary (59.4%) and conventional farming system in Serbia (60.3%) than at the conventional farming system in Hungary (52.5%). In spelt wheat, starch content ranged from the 49.7% found at the conventional farming system in Hungary to 62% at the organic farming system in Hungary. In both species, conventionally grown varieties from Serbia had higher starch content (56.3% for bread and 60.3% for spelt wheat) than conventionally grown varieties from Hungary (54% for bread and 55.6% for spelt wheat).

The analysis of variance showed significant differences between wheat species, varieties, growing seasons and farming systems based on bioactive compound, protein and starch content (Table 4). Bread wheat had significantly higher total and water-extractable arabinoxylan, β-glucan, fructan and starch content than spelt wheat. Bread wheat variety ‘Recital’ had the highest total (18.00 mg/g) and water-extractable (7.83 mg/g) arabinoxylans. The lowest total and water-extractable arabinoxylan content was found in ‘Estevan’ and ‘Apache’ varieties. The β-glucan content was the highest in ‘Pobeda’ (7.84 mg/g) and the lowest in ‘Recital’ (6.87 mg/g). ‘Estevan’ had the highest alkylresorcinol content (547.18 µg/g), whereas the bread wheat varieties ‘Pobeda’, ‘Recital’ and ‘Balkan’ had the highest fructan content. Among the analyzed varieties, the highest protein and starch content was measured in ‘Estevan’ and ‘Recital’, respectively.

Among spelt wheat varieties, total and water-extractable arabinoxylan did not vary significantly (Table 4). Spelt varieties did not differ in β-glucan and alkylresorcinol content, except for the varieties ‘Baulander Spelz’ and ‘Ostro’ in β-glucan and ‘Baulender Speltz’ and ‘Rouquin’ in alkylresorcinol. The fructan content was the highest in ‘Baulender Speltz’ (0.91%), ‘Ostro’ (1%) and ‘Oberkulmer-Rotkorn’ (0.88%). ‘Ostro’ (15.11%) and ‘Oberkulmer-Rotkorn’ (15.05%) had the highest protein content, whilst the starch content was the highest in ‘Schwabenkorn’ (56.66%). The growing season influenced all the traits except fructan and starch content in bread wheat and β-glucan and fructan content in spelt. Differences among farming systems and countries were significant for all the traits except for the total arabinoxylan content in bread wheat and fructan content in spelt wheat.

### 3.2. Differences in the Effect of the Variety (Genotype) and the Environment (Farming System and Country) on Bioactive Compound, Protein and Starch Content of Bread and Spelt Wheat

**Figure 2:**
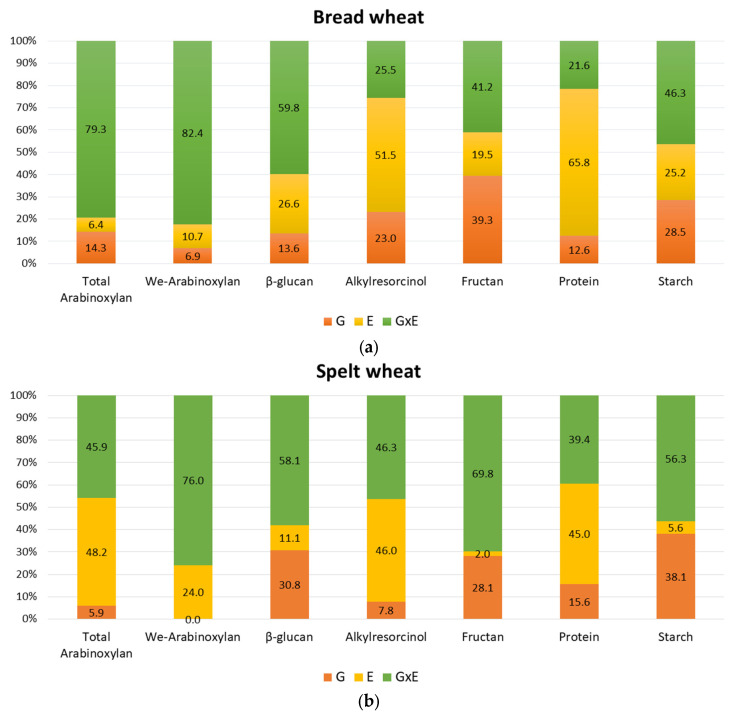
Column charts of the relative contribution of genotype (G), environment (E—3 farming systems) and genotype × environment interaction (G×E) to the total variance of the bioactive compound, protein and starch content of bread (**a**) and spelt (**b**) wheat.

The G×E interaction determined more than 70% of the variance of total and water-extractable arabinoxylan in bread wheat (Figure 2a). A considerable effect of the G×E interaction was also found for β-glucan, fructan and starch content determining more than 40% of the total variance. A great environmental effect (farming system/country/growing season) was observed for alkylresorcinol and protein content (>50%). A large G×E interaction effect (˃45%) was found for all traits in spelt, except protein content (Figure 2b). The effect of the environment (farming system/country/growing season) was the greatest on total variance of protein content.

### 3.3. Correlations of Bioactive Compound, Protein and Starch Content in Bread and Spelt Wheat

**Figure 3:**
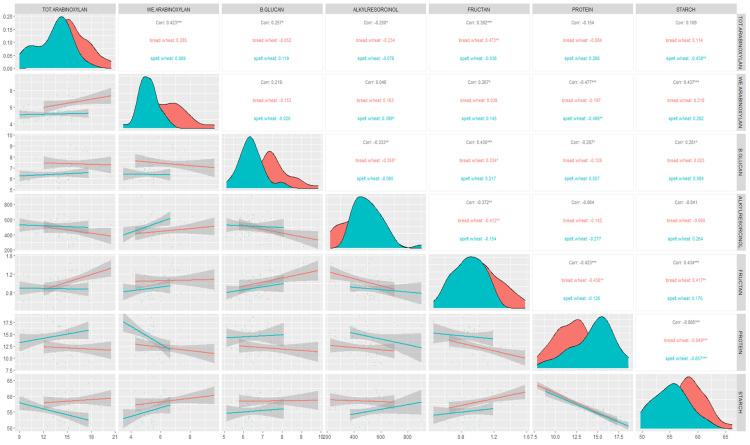
Correlations between the bioactive compound, protein and starch content for bread and spelt wheat. Above the diagonal: the Pearson’s correlation coefficients for bread (red) and spelt (blue) wheat and combined (gray); diagonal: the distributions of each trait for bread (red) and spelt (blue) wheat; below the diagonal: scatter plots with linear regression lines for bread (red) and spelt (blue) and their confidence intervals (gray areas). Correlation coefficients significant for *p* < 0.001 are marked with ***, those significant for *p* < 0.01 with ** and *p* < 0.05 with *. The correlation coefficients that are not marked are not significant.

According to the Pearson’s coefficients, significant positive correlations were found between total and water-extractable arabinoxylan, β-glucan and fructan content in both species combined (Figure 3). Water-extractable arabinoxylan showed positive correlations with fructan and starch content, while β-glucan correlated positively with fructan and starch content. Significant positive correlations were also found between fructan and starch content. On the other hand, for both species combined, significant negative correlations were found for total arabinoxylan and alkylresorcinol content as well as water-extractable arabinoxylan and protein content. Significant negative correlations were found between β-glucan and protein and between β-glucan and alkylresorcinol content. Fructan content was found to be in significant negative correlation with alkylresorcinol and protein content. Starch and protein content also showed significant negative correlation.

In bread wheat, significant positive correlations were found for total arabinoxylan and fructan content, β-glucan and fructan content and fructan and starch content. Conversely, significant negative correlations were found between β-glucan and alkylresorcinol content, fructan and alkylresorcinol content, fructan and protein content and protein and starch content. In spelt wheat, significant positive correlation was found for water-extractable arabinoxylan and alkylresorcinol content, whilst significant negative correlations were found between total arabinoxylan and starch content, water-extractable arabinoxylan and protein and protein and starch content.

### 3.4. Principal Component Analysis of Bread and Spelt Wheat Grown under Conventional and Organic Farming Systems in Hungary and Serbia

**Figure 4:**
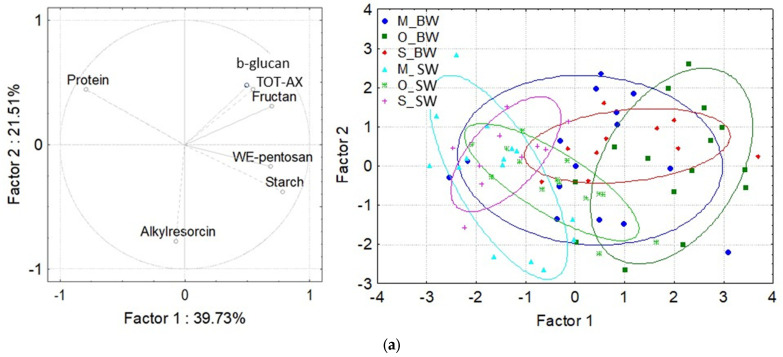

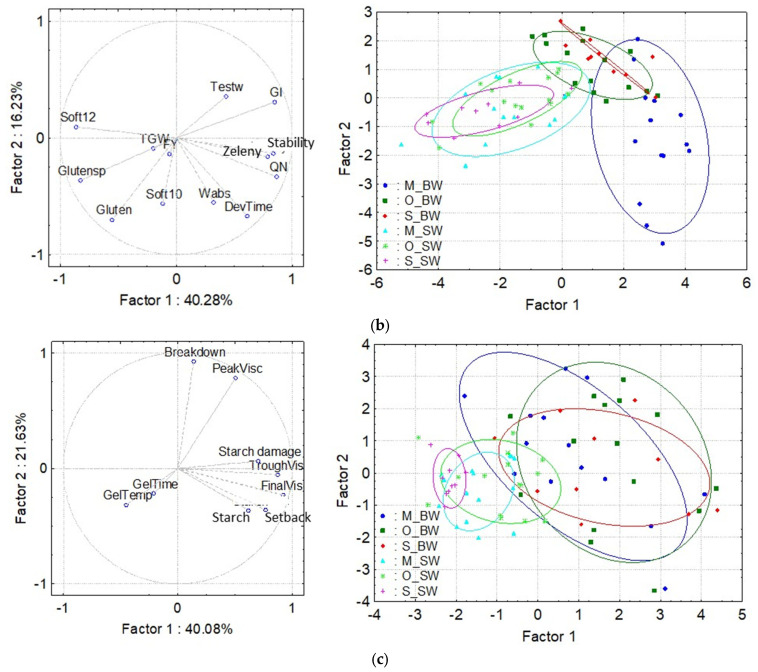
Principal component analysis of bread and spelt wheat varieties based on (**a**) bioactive compound, protein and starch content, (**b**) physical and quality traits and (**c**) starch-related components. M—conventional farming system in Martonvásár, Hungary, O—organic farming system in Hungary, S—conventional farming system in Serbia. BW—bread wheat, SW—spelt wheat. TOTAX—total arabinoxylan, WEAX—water-extractable arabinoxylan, Soft10, 12—dough softening after 10 and 12 min, respectively, Glutensp—gluten spread, TGW—thousand kernel weight, Testw—test weight, FY—flour yield, Wabs—water absorption of the flour, DevTime—dough development time, QN—Farinograph quality number, GI—gluten index, Stability—dough stability, Zeleny—sedimentation volume, PeakVisc—viscosity at peak, TroughVisc—remaining viscosity after breakdown, FinalVisc—viscosity after setback at the end of the temperature profile, Setback—increasing of the viscosity when cooling, Breakdown—decreasing of the viscosity at high temperature, Pasting Time—the time when gelatinization starting, Pasting Temperature—the temperature at which gelatinization is starting, Starch Damage—measured by amperometric method. Red—Serbia, Green—Organic, Blue—Martonvásár, light color—spelt, dark color—bread wheat.

PCA analysis based on combining bioactive compound, protein and starch content clearly separated organically grown bread wheat from conventionally grown spelt wheat from Hungary and Serbia (Figure 4a). The first PC accounted for 39.73% of the total variation and was determined by β-glucan, total and water-extractable arabinoxylan, protein, fructan and starch content. The second PC explained 21.51% of the total variation and was determined by the alkylrezorcinol content. Organically grown bread wheat varieties from Hungary and conventionally grown bread wheat varieties from Serbia with high values of total and water-extractable arabinoxylan, fructan and starch content were clustered on the right side of the biplot. On the other side of the biplot, the conventionally grown spelt wheat varieties from Serbia with high protein content and low alkylresorcinol content were clustered towards the protein vector in the upper left-hand quadrant. Sligtly below this group in the middle of the left side of the biplot were positioned the organically grown spelt wheat varieties from Hungary with high alkylresorcinol and low protein content, towards the alkylresorcinol vector. On the same side of the biplot, the conventionally grown spelt wheat varieties from Hungary with high protein and alkylresorcinol content spread towards both protein and alkylresorcinol vectors. Conventionally grown bread wheat from Hungary did not separate from any group as it contained high variation for the majority of analyzed traits.

Protein-related quality traits also differed at the different sites and in different species (Figure 4b). The first and the second PC accounted for 40.28% and 16.23%of the total variation, respectively, and partly separated the bread wheat varieties on the right-hand side from the spelt varieties on the left-hand side of the biplot. The organically grown bread wheat varieties from Hungary and conventionally grown bread wheat varieties from Serbia with high values for test weight and gluten index grouped on the upper right quadrant near the vectors for these traits. On the same side of the biplot, the conventionally grown bread wheat varieties from Hungary were located near the vectors for gluten index, water absorption, dough stability and development time, quality number and sedimentation volume. On the opposite side of the biplot, spelt wheat varieties with high values of gluten content, gluten spread and dough softening were positioned near their respective vectors.

The PCA that was based on the starch-related components distinguished bread wheat from spelt wheat varieties to a large extent (Figure 4c). The first PC was defined by most of the starch-related components and accounted for 40.08% of the total variation. The second PC acounted for 21.63% of the total variation and was determined by the peak viscosity and the breakdown of the viscosity. The PCA clustered spelt wheat varieties with low gelatinization time and temperature on the left side of the biplot, opposite to conventionally and organically grown bread wheat varieties with better pasting properties clustered on the right side of the biplot.

## 4. Discussion

### 4.1. Bioactive Compound, Protein and Starch Content and Quality in Bread and Spelt Wheat Species

Extensive cultivation, coupled with high yields, reduced spike-shattering and free-threshing, placed bread wheat among the most cultivated *Triticum* species. Although at some point the farming of spelt over bread wheat was neglected, at present, the production of spelt is extended, which can be attributed to its preferable health-related properties, greater amenability to sustainable agriculture [43] and good adaptation to low-input and organic farming systems [44] compared to bread wheat. Those findings prompted us to explore possible differences in health-related compounds of five modern bread—and five modern, and older, spelt—wheat varieties.

Even though spelt and bread wheat have a very similar distribution of dietary fiber over the kernel cell walls [45], their amounts differ. The results from our study showed significantly higher amounts of dietary fiber, including arabinoxylan, fructan and β-glucan, in bread wheat than in spelt wheat. Some studies have reported that bread wheat generally has higher amount of total dietary fiber [46,47], while other studies revealed no significant differences among these two hexaploid species [48] although a slightly higher amount was seen in bread wheat. From total fiber, the amount of total arabinoxylan showed higher values in bread than in spelt wheat [25,26,45]. Moreover, bread wheat exhibited more fructan compared to spelt wheat [26,45]. While some authors reported more β-glucan in bread wheat [25,49], others found similar amounts in spelt and bread wheat [45]. Considering all this, the coat of bread wheat might possesses a stronger protective function than spelt wheat. Contrary results came from Gebruers et al. [25], who found no differences in arabinoxylan—including total and water-extractable—and β-glucan content among bread and spelt wheat. The reason for these conflicting results could be attributed to the small number of spelt samples (*n* = 5) compared to a larger bread wheat sample size (*n* = 151); the sample sets were unbalanced. The significantly higher amount of arabinoxylan in bread than in spelt wheat found in our study could be explained by differences in the pericarp layers. Escarnot et al. [45] observed, by microscopic analysis, that the bread wheat pericarp has three layers whereas the spelt wheat pericarp has one cell layer. As arabinoxylan is predominant in the pericarp layer, accounting for 47% [50], a higher content of arabinoxylan can be found in the pericarp layer of bread wheat. Antoine et al. [51] found variation within bread wheat as well, as hard wheat had higher bran yield and arabinoxylan content, and less β-glucan in the pericarp with a denser aleurone layer, than soft wheat [52]. Recently, the staining of β-glucan in bread and spelt wheat kernel cross-sections revealed higher intensity in bread wheat, confirming higher β-glucan content [26]. Moreover, the staining allowed researchers to report that β-glucan was confined mostly in the aleurone and its sublayer in spelt wheat and in the endosperm of bread wheat [26]. As endosperm accounts for 80–85% of the wheat kernel [53], higher values of β-glucan found in our study could be explained by the localization of this polysaccharide. Fructan, which is localized in both the endosperm and the bran, is found in much higher amounts in the bran fraction than in the endosperm, and concretely in the aleurone layer [54]. As spelt has a larger share of aleurone layer in the kernel than bread wheat [55], our findings on higher fructan content in bread wheat than in spelt wheat are contradictory. As the majority of spelt wheat varieties used in our study were soft [56,57], while the bread wheat varieties were hard [58,59], histological differences in kernels of the two wheat species could be responsible for the higher arabinoxylan, β-glucan and fructan content of bread wheat.

The literature findings about the antioxidant capacities related to the alkylresorcinol content in spelt and bread wheat are contradictory. While some authors reveal no significant differences [60], others did. Andersson et al. [61] found significantly higher values of alkylresorcinol in spelt than in bread wheat, while Ziegler et al. [62] reported the opposite. Several researchers reported better antioxidant capacity in spelt than in bread wheat [49,63], which is in accordance with our results. Significant differences between the two species could be the result of the variations in the relative homologues of the alkylresorcinol composition in our study. Alkylresorcinol represents a natural homologous series of phenolipid antioxidants, the antioxidant capacity of which is in positive relation with the length of the alkyl chain to the point of the hydrophobicity threshold [64]. In wheat, alkylresorcinol homologues ranging from C17 to C25 chain length are present in high concentrations [65]. Elder et al. [66] found the optimum antioxidant activity at an intermediate alkyl chain length (C21:0), after which the activity declined. By characterizing the alkylresorcinol homologue profiles in spelt and bread wheat, several studies [9,10,61] found that the predominant homologue was C21:0 in both species, which represented almost half of the total alkylresorcinol content, followed by C19:0 and C23:0. By comparison of those three homologues in bread and spelt wheat, Pedrazzani et al. [9] reported higher values for C21:0 and C19:0 in spelt wheat than in bread wheat varieties. Additionally, significantly higher value was observed for the ratio of the two dominant homologues (C21:0/C23:0) in spelt compared to bread wheat [9]. Thus, spelt has stronger antioxidant capacity than bread wheat. Although Ziegler et al. [60] did not find significant differences in the total alkylresorcinol content, differences were found among the homologues. While the ratio of C19:0 was higher in bread—and C23:0 in spelt—wheat, the C21:0 homologue did not differ among species. Nevertheless, differences could be attributed to the higher percentage of aleurone layer (including testa) in soft wheat than in hard wheat [51].

Significant positive correlations between total and water-extractable arabinoxylan and β-glucan, as well as fructan and β-glucan, were found in our study for both species, and this is in agreement with other studies [8,25]. In our study, significant negative correlations were found between total and water-extractable arabinoxylan and alkylresorcinol, as well as β-glucan and alkylresorcinol, which is consistent with other studies [8]. This can be explained by the fact that they are accumilated in different tissues of the bran. The negative correlation between the total arabinoxylan and starch content in bread wheat found in our study, and by Gebruers et al. [67], suggests that bread wheat varieties with higher dietary fiber content gravitate towards lower caloric value.

The largest kernel fraction comes from the endosperm, in which starch is the predominant compound [2]. Starch is the main source of dietary carbohydrates and its molecular structure determines the functional properties of the end-use products. Although having the same ploidy level, bread and spelt wheat varieties in our study showed significant variations in starch content, with bread wheat having more starch. Some previous studies reported opposite findings [68], while others reported no significant differences [48]. The higher starch content in bread wheat in our study can be related to variations in kernel texture among species. Several authors reported a significantly higher hardness index in bread than in spelt wheat [48,69]. Additionally, by analyzing the microstructure of spelt and bread wheat kernels, it was shown that starch granules of spelt wheat were less tightly nested into the inner structure of the protein matrix than the bread wheat starch granules [48], which can be a characteristic of species and can also explain differences in the amount of starch between spelt and bread wheat.

Protein is the second main component of wheat grain. In our study, spelt wheat varieties showed significantly higher protein content than bread wheat varieties, which is in accordance with the majority of the literature results [27,47,63,68,70]. For example, by analyzing the protein content with three different methods, such as near-infrared spectroscopy, the Dumas method and reverse-phase high-performance liquid chromatography, Call et al. [71] revealed significantly higher protein content in spelt than in bread wheat. One of the reasons for the higher protein content of spelt can be explained by the different kernel size distribution. Namely, by comparison of the kernel size distribution of spelt and bread wheat, Kulathunga et al. [48] revealed that spelt possessed significantly more large kernels, while the dominant kernels in bread wheat were medium-sized kernels. No differences were observed in small-size kernels. Based on these researches, more protein content is expected in spelt wheat. Other reason could be attributed to the higher thousand kernel weight [48,68] and nitrogen content [63] in spelt wheat.

By linking protein-related traits and starch pasting properties, a similar pattern was observed in our study. Namely, spelt wheat was characterized by higher flour yield and thousand kernel weight, dough softening, gluten content and gluten spread, while bread wheat had higher test weight, gluten index, farinograph quality number, water absorption, dough development time, dough stability and Zeleny sedimentation volume. As an indicator of milling value, spelt is expected to have higher flour yield, as it has higher thousand kernel weight [48,72], kernel length and thickness than bread wheat [73]. Although the essential criterion responsible for breadmaking quality is good gluten content and quality [55], which is present in higher amounts in spelt than bread wheat [74,75], many authors report on the poor quality of spelt wheat [76,77]. Namely, spelt gluten is characterized by high dough softening that results in significant dough extensibility [56]. Moreover, higher gluten spread, related to a weaker gluten structure, is usually observed in spelt wheat [78,79]. The poor quality of spelt wheat could also be demonstrated by the significant negative correlations found between dough development time and dough softening as well as dough softening and stability [80]. The results from our study confirmed previous studies related to the poor quality of spelt wheat. Opposite to spelt, better quality is found in bread wheat, which is confirmed by higher test weight, one of the most often used wheat quality indices. Test weight is influenced by abiotic and biotic factors and some morphological characteristics, such as kernel shape and density. Higher test weight in bread wheat is in accordance with Kulathunga et al. [48] and is a result of the variations in physical properties of spelt and bread wheat kernels [73]. The gluten index and sedimentation volume are one of the most common methods used for the evaluation of the gluten properties [81]. Better bread wheat gluten quality, represented by higher gluten index and sedimentation volume [82], indicate higher gluten strength. Furthermore, good breadmaking quality was confirmed by the better rheological properties of the dough presented by water absorption, dough development time and stability [27]. All differences in the rheological properties of the gluten in the two hexaploid species could be related to the protein composition. Glutenins prevail in bread wheat, while gliadins prevail in spelt [79]. As glutenins are polymeric proteins, they absorb more water and need a longer time to develop dough, and as a result, the stability of the dough is better and higher bread volume is gained [27]. These associations are supported by the significant negative correlation found between bread volume and dough softening [83]; significant positive correlations between polymeric glutenins, water absorption, dough development time and stability; and significantly negative correlations between monomeric gliadins, water absorption, dough development time and stability [27]. In addition, the higher farinograph quality number of bread wheat was also related to better rheological properties and confirmed by a significant positive correlation between water absorption, dough development [84] and stability time and quality number [83]. The relationship between bioactive compounds, especially water-extractable arabinoxylan, and quality is reflected through increasing viscosity [85], water absorption capacity [86] and dough development time [87].

One of the main factors contributing the most to the functional properties of cereals are the molecular weight of the amylopectin and the distribution of amylopectin structural units [88]. By combining starch pasting properties, our results confirmed the work of Brandolini et al. [68] who found better pasting properties of bread than spelt wheat. Those results were further corroborated by the significant positive correlation of starch content and peak viscosity, breakdown, final viscosity and setback properties. The variations in pasting properties in our study can arise from variations in the molecular structure of spelt and bread wheat starches. More precisely, spelt and bread wheat differ in starch polysaccharide chains, with bread wheat exhibiting much higher molecular weight and broader molecular weight distribution. Moreover, spelt amylopectin contains twice as much of the shortest, A-type chains, as well as B-type, than bread wheat amylopectin [89]. Those variations could be important when studying rheological properties of different flours, since small starch granules have higher gelatinization temperatures and lower enthalpy compared to larger granules [90].

### 4.2. The Response of Genotype and the Exceptional Characteristics of the Varieties

The response of genotype (G), environment (Hungary and Serbia, growing season, farming system; E) and the G×E interaction contributed to the variation in the bioactive compound, protein and starch content in different manners, depending on wheat species. By comparing the effect of genotypes on total dietary fiber, Escarnot et al. [46] found that the genotype was a source of variation in spelt but not in bread wheat. According to Gebruers et al. [67], both the genotype and the environment explained approximately 30%, and the G×E interaction 20%, of the total variability. Genotype had the most profound effect on total and water-extractable arabinoxylan [67]. Finnie et al. [91] reported that the main source of variation for water-extractable arabinoxylan was genotype, while the genotype and environment shared equal contribution to the total arabinoxylan. Other authors reported that the variation in total and water-extractable arabinoxylan was mostly explained by the environment, followed by the genotype, and that the G×E interaction was also a significant source of variation [92]. The large contribution of the genotype to the total variance could be the result of the high heritability of the total [93] and water-extractable arabinoxylan [94]. The low impact of the genotype on the total arabinoxylan content found in our study is congruent with the results of Dornez et al. [95] who considered this parameter not suitable for breeding purposes. The same results were confirmed in durum wheat, where the environment was the main source of variation for both total and water-extractable arabinoxylan [96], which means that the environmental conditions can considerably determine the accumulation of arabinoxylan.

The β-glucan content is mostly influenced by the genotype in wheat [97], whereas the environment and the G×E interaction equally contributed to the variation [67]. In other studies, the G×E interaction’s contribution was higher than the contribution of the environment [97]. A significant genotypic variation for fructan content was also reported in bread wheat, and was explained by the high heritability of this trait [98]. In our study, the strongest effect of the G×E interaction on β-glucan and fructan content in both species could be the result of the interaction with other factors, such as the agricultural input and soil properties [67]. Research on the content and the composition of alkylresorcinol showed a significant influence of genotype, environment and their interaction [99], with genotype having the highest contribution to the variance [49]. Although highly heritable [62], the alkylresorcinol content is also affected by environmetal [99] and meteorological conditions [9]. Finally, all variations in bioactive compounds presented in our study can be explained by different expression levels of the genes in different genotypes resulting in different grain dietary fiber contents in bread wheat [100].

In spite of the strong influence of genotype on protein [70] and starch content [101], the environment also has a significant effect on both components [102]. Labuschagne et al. [103] reported that the environment contributed the most to the protein and starch content, followed by the genotype for protein and the G×E interaction for starch content. Those results were recently confirmed in bread wheat [104]. In our study, the environment and the G×E interaction contributed the most to the variance of protein and starch content in both species, respectively. These results suggest that, except for a strong genetic background, in a specific environment and under a specific microclimate, the abiotic factor greatly shapes certain traits. Larger environmental differences in our study made it possible to detect even small genotypic differences in protein content.

In our study, the following bread wheat varieties are suitable for breeding as rich sources of bioactivity. These are ‘Recital’ with the highest arabinoxylan and starch content, ‘Pobeda’ with the highest β-glucan and fructan content and ‘Estevan’ with the highest antioxidant capacity and protein content. Among spelt wheat varieties, ‘Oberkulmer-Rotkorn’ had the highest amount of arabinoxylan and β-glucan content and high antioxidant capacity, and the lowest starch content. ‘Rouquin’ had the highest alkylresorcinol content. It is documented that type 2 diabetes is commonly diagnosed in people whose diet is based on starch, such as Asians [105], and as the food industry is constantly exploring natural antioxidant sources for food conservation [106], the flour of ‘Oberkulmer-Rotkorn’ and ‘Rouquin’ varieties could be a possible solution when applied as a supplement in the production of various food products. Due to its high softening [80], ‘Oberkulmer-Rotkorn’ could be used only as a supplement in breadmaking. The high antioxidant capacity in ‘Oberkulmer-Rotkorn’ is also confirmed by previous studies [60,62]. Biel et al. [107] found by comparing three new breeding strains and the ‘Oberkulmer-Rotkorn’ variety that ‘Oberkulmer-Rotkorn’ also has the highest β-glucan and protein content. Next to ‘Oberkulmer-Rotkorn’, the varieties ‘Ostro’—with the highest fructan and protein content—and ‘Schwabenkorn’—as a rich source of starch—could also be used in breeding programs. In our study, the highest protein content was found in ‘Ostro’ and ‘Oberkulmer-Rotkorn’, the latter being a parent in the former’s pedigree. The high protein content in the ‘Ostro’ variety is confirmed by Bonafacia et al. [108], but ‘Oberkulmer-Rotkorn’ and ‘Schwabenkorn’ were also reported to have high protein and gluten content, as well as good sedimentation index and baking values. These are possibly useful sources of genes for improving the nutritional value by crop breeding to develop healthy grains [75].

### 4.3. Bioactive Compound, Protein and Starch Content and Quality in Different Farming Systems

There is some evidence that organically grown wheat has more dietary fiber [17,109] and lower antioxidant capacity than conventionally grown wheat [110], but other investigations showed no difference in bioactive compound content [111,112]. Some researchers found more soluble fiber in organically grown wheat [113], while some found such in conventionally grown wheat [105]. Slightly higher β-glucan content was found in organically than in conventionally grown wheat [114]. On the other hand, better antioxidant capacity and higher alkylresorcinol content [112] were found in organically grown durum wheat [115].

In our study, significant differences between conventional and organic farming systems were found for water-extractable arabinoxylan, β-glucan, alkylresorcinol, protein and starch content in both species. Differences among farming systems were also found for fructan in bread wheat and for total arabinoxylan content in spelt.

Significantly higher total arabinoxylan content in spelt wheat in our study can be explained by the fact that spelt wheat successfully grows in organic and low-input fields [44] where it can express its full potential.

The significantly higher β-glucan content in organically grown bread wheat observed in our study corroborates a previous study [116]. As organic farming does not use any synthetic chemicals, it is possible that synthetic chemicals intefere in the biosynthetic pathway of β-glucan, which acts as an assimilate buffer. A decrease in β-glucan in response to fungicide in barley was demonstrated by Dickin et al. [117]. On the other hand, the significantly lower β-glucan content of spelt wheat at the organic growing site in our study might be the result of the different nitrogen rates [118]. Lower protein content [119] could also be the result of the lack of mineral nitrogen fertilizers used in organic farming. Significantly higher protein content was reported in varieties treated with mineral NPK in comparison to the same varieties treated with organic fertilizers [120]. A higher starch content was observed in organically grown wheat species, which might be explained by the significant negative correlation between soil nitrogen and total starch [102]. More fructan in bread wheat in our study can be attributed to a significant, negative correlation between fructan and N-rich compounds, nitrate reductase activity and N metabolism-related transcript levels found in the study by Vicente et al. [121]. They highlighted that the accumulation of fructans could hinder photosynthesis and be regulated by N assimilation to adjust the metabolic status to the requirements of the crop. Morcuende et al. [122] reported that fructan biosynthesis in barley was upregulated by sugar signaling and downregulated by nitrogen.

Generally, it is argued that the arabinoxylan accumulation is influenced by meteorological conditions, primarily water availability. Higher amounts of rainfall result in higher amounts of total [92] and water-extractable arabinoxylan in bread [95,97] and durum wheat [96]. Beside meteorological conditions, other factors, such as soil type and agronomical input, also affect arabinoxylan content [97]. Opposed to this finding, more water-extractable arabinoxylan content was found at the conventional farming system in Hungary in our study, in both species, where the precipitation in the full growing season and in the last 100 days before harvest was lower than in Serbia. This can be explained by the effect of heat and drought stress resulting in increased total and water-extractable arabinoxylan content in wheat grain, but timing of the stress is also important [123]. In the last 100 days before harvest there was a pronounced drought in Hungary, especially in the 2019/2020 and 2020/2021 growing seasons, and several days where heat was higher than 30 °C. At the same time, Serbia had lower temperatures and more precipitation. Andersson et al. [99] found the weather condition to be crucial for the accumulation of alkylresorcinol; thus, high precipitation resulted in its lower quantities especially during plant development and grain-filling periods. In Serbia, the amount of precipitation was twice as much as the amount in Hungary in the last 100 days before harvesting over the same period of the 2019/2020 growing season. More protein content was found in seeds grown at the conventional site in Hungary in comparison to the conventional site in Serbia in bread wheat, which could be the result of more N being used through the NPK combined fertilizer, as higher available nitrogen levels lead to higher protein content in bread wheat [124]. Higher amounts of precipitation and fewer days above 30 °C in the last 100 days before harvest in Serbia resulted in significantly higher starch content in comparison to Hungary, which is in accordance with other studies that reported a positive correlation of starch content to precipitation [101] and a negative correlation with the number of days above 30 °C after anthesis [125]. The latter results from the decreasing of amylopectin content of starch.

## 5. Conclusions

Comparison of the compositional traits of spelt and bread wheat genotypes confirmed the significant differences between the arabinoxylan, β-glucan, alkylresorcinol, fructan, protein and starch content of the two species. Accordingly, spelt had significantly higher protein and alkylresorcinol content, but all the other components were present in lower quantities in spelt than in bread wheat. The higher protein content and antioxidant capacity of spelt has already been confirmed by other authors as well. Both have nutritional and health-related benefits. The lower fructan content of spelt makes it a possible target for studies aiming at the production of food for FODMAP-sensitive patients. The significantly lower total and soluble fiber content of spelt could be beneficial in animal feeding.

Farming system (conventional, organic) significantly affected all studied parameters, except the total arabinoxylan content of bread wheat and the fructan content of spelt wheat. Organic spelt had significantly higher starch, fiber and alkylresorcinol content but lower β-glucan and protein content than conventionally grown spelt. Organic bread wheat showed the same, but more pronounced, differences compared to conventional sites, except for the protein content, which was lower at Serbian and higher at Hungarian conventional sites. The fructan content of organic bread wheat was higher than the conventionally grown ones. The lower protein generally found under the organic site could be a possible explanation of the weaker breadmaking quality (lower dough strength), which is also a characteristic of spelt compared to bread wheat. Spelt showed lower test weight, gluten index, farinograph quality number, water absorption, dough development time, dough stability and Zeleny sedimentation volume. Starch properties such as peak viscosity, breakdown, final viscosity, trough viscosity and setback properties were also lower in spelt.

The variety had significant effect on all compositional traits in bread wheat, while only β-glucan and fructan content was affected by spelt varieties. This might be the result of the low variability found in spelt properties, although neither of the spelt genotypes contained the wheat genome, and the strong effect of G×E with a lower effect of G.

From spelt wheat varieties, ‘Oberkulmer-Rotkorn’ had the highest arabinoxylan, β-glucan and starch content, with high values of protein, alkylresorcinol and fructan content as well. It had high protein content under conventional (above 14%) as well as organic farming (above 13%), and it is considered to have a greater potential for organic, high-quality, milling spelt wheat production.

Overall, our results demonstrated that the environmental conditions, farming systems and wheat species contribute to the variations in the amount of the bioactive compounds, protein and starch content in different manners. These findings on the composition of individual bioactive compound, protein and starch content in specific varieties and wheat species can serve as a starting point for breeders to breed new varieties with improved dietary fiber content, antioxidant capacity and, consequently, improved nutritional value.

## Figures and Tables

**Table 1:** The country of origin, the year of release and the pedigree of bread and spelt wheat varieties analyzed in this research according to the Genetic Resources Information System for Wheat and Triticale (GRIS) database.

Species/Variety	Pedigree	Origin	Year
*T*. *aestivum* L. subsp. *aestivum*			
Apache	Axial/NRPB-84-4233	FRA	1998
Balkan	Bačka/Bez1//Miron808/3/NS433/4/Skor35	SRB	1979
Estevan	Capo/SE-24090	DEU	2009
Pobeda	Sremica/Balkan	SRB	1990
Recital	Mexique-267(R-267)/9369	FRA	1986
*T*. *aestivum* L. subsp. *spelta*			
Baulander Spelz	Geiberger Spelz	DEU	1926
Ostro	Oberkulmer-Rotkorn/Steins-Roter-Tiroler	CHE	1978
Rouquin	Lignée-24/Ardenne//Altgol	BEL	1979
Schwabenkorn	(S)LV	DEU	1988
Oberkulmer-Rotkorn	(S)LV-CHE	CHE	1948

BEL—Belgium; CHE—Switzerland; DEU—Germany; FRA—France; SRB—Serbia.

**Table 2:** Developing and environmental conditions of different farming systems during three growing seasons (2018/2019, 2019/2020 and 2020/2021) in Hungary and two growing seasons (2018/2019 and 2019/2020) in Serbia.

Developing Conditions		Hungary	Serbia
Position	geographic coordinates	47°18′ N,	45°20′ N,
		18°47′ E	19°51′ E
	Altitude (m)	115	84
Developing parameters	previous crop: conventional/organic	2019/20: facelia/facelia 2020/21: oil radish/buckwheat 2021/22: oil radish/buckwheat	2018/19: soybean 2019/20: soybean
	sowing density wheat/spelt (seed/m^2^)	500/280	550
Soil parameters	soil type	chernozem	chernozem
	pH (KCl)	7.25	7.41
	humus (*m*/*m*%)	2.8	2.6
	P_2_O_5_ (mg/kg)	210	208
	K_2_O (mg/kg)	210	176
	average nitrogen input through nitrogen, phosphorus and potassium (NPK) fertilizer (conventional) per year (active ingredient, kg/ha)	120	100

**Table 3:** Meteorological conditions for the three growing seasons in Hungary and two growing seasons in Serbia.

		2018/2019		2019/2020		2020/2021
		Hungary	Serbia	Hungary	Serbia	Hungary
full season	Growing period (days)	279	264	288	274	288
	Cumulative precipitation (mm)	365.6	435.3	382.3	473.5	300.5
	Mean temperature (°C)	9.3	11.5	9.48	11.6	8.81
	Absolute min temperature (°C)	−14.4	−16.3	−8.8	−6.6	−11.6
	Absolute max temperature (°C)	36.0	35.0	33.3	34.4	37.4
last 100 days	Cum. precipitation in the last 100 days before harvest (mm)	225.0	265.4	132.5	226.5	116.1
	Mean temperature in the last 100 days (°C)	17.1	17.5	16.3	18.7	16.3
	Absolute min temp in the last 100 days (°C)	−0.7	−0.9	−3.6	−5.4	−6.5
	Absolute max temp in the last 100 days (°C)	36.0	35.0	33.3	34.4	37.4
abs. min–max	No of days with Tmin ≤ 0 °C	90	75	90	52	85
	No of days with Tmin ≤ −10 °C	6	3	0	0	3
	No of days with Tmax ≥ 25 °C	42	38	36	45	40
	No of days with Tmax ≥ 30 °C	16	17	6	8	18
	No of days with Tmax ≥ 35 °C	1	1	0	0	1

**Table 4:** Bioactive compound, protein and starch content depending on wheat species, variety, growing season and farming system.

	Total	Water Extractable					
Trait	Arabinoxylan	Arabinoxylan	β-Glucan	Alkylresorcinol	Fructan	Protein	Starch
	mg/g	mg/g	mg/g	µg/g	%	%	%
Species	<0.0001 ***	<0.0001 ***	<0.0001 ***	0.004 **	<0.0001 ***	<0.0001 ***	<0.0001 ***
*T. aestivum* L. subsp. *aestivum*							
Variety	<0.0001 ***	<0.0001 ***	0.0300 *	<0.0001 ***	<0.0001 ***	0.0020 **	<0.0001 ***
Apache	14.73 ^a,b^	5.66 ^a^	7.13 ^a,b^	509.68 ^c,d^	1.01 ^a,b^	10.81 ^a^	58.81 ^b^
Balkan	16.08 ^b,c^	6.81 ^b^	7.64 ^a,b^	406.17 ^a,b^	1.08 ^b^	12.56 ^b^	57.81 ^b^
Estevan	13.49 ^a^	5.87 ^a^	7.27 ^a,b^	547.18 ^d^	0.80 ^a^	13.55 ^c^	56.36 ^a^
Pobeda	16.21 ^c^	6.73 ^b^	7.84 ^b^	366.29 ^a^	1.23 ^b^	12.35 ^b^	57.99 ^b^
Recital	18.00 ^d^	7.83 ^c^	6.87 ^a^	443.62 ^b,c^	1.20 ^b^	10.78 ^a^	61.93 ^c^
Average	15.70 ^B^	6.58 ^B^	7.36 ^B^	454.87 ^A^	1.06 ^B^	12.04 ^A^	58.49 ^B^
Growing season	<0.0001 ***	<0.0001 ***	0.0020 **	<0.0001 ***	0.0770 n.s.	0.0010 ***	0.0770 n.s.
2018/2019	14.96 ^a^	6.18 ^a^	7.75 ^b^	372.18 ^a^	1.04 ^a^	12.87 ^c^	57.82 ^a^
2019/2020	17.12 ^b^	6.19 ^a^	7.34 ^a,b^	457.75 ^b^	1.14 ^a^	12.00 ^b^	58.23 ^a^
2020/2021	14.69 ^a^	7.78 ^b^	6.82 ^a^	574.87 ^c^	0.98 ^a^	10.86 ^a^	59.88 ^b^
Farming system	0.7980 n.s.	<0.0001 ***	0.0140 *	0.0120 *	<0.0001 ***	<0.0001 ***	<0.0001 ***
Hungary							
Conventional	16.12 ^a^	7.05 ^b^	7.03 ^a^	483.56 ^b^	0.92 ^a^	13.72 ^c^	56.26 ^a^
Organic	15.44 ^a^	6.92 ^b^	7.73 ^b^	477.01 ^b^	1.16 ^b^	10.54 ^a^	59.39 ^b^
Serbia							
Conventional	15.46 ^a^	5.36 ^a^	7.27 ^a,b^	381.48 ^a^	1.10 ^a,b^	11.93 ^b^	60.28 ^b^
*T. aestivum* L. subsp. *spelta*							
Variety	0.2560 n.s.	0.6410 n.s.	<0.0001 ***	0.0550 n.s.	<0.0001 ***	0.1220 n.s.	0.4340 n.s.
Baulander Spelz	12.73 ^a^	5.10 ^a^	4.98 ^a^	454.93 ^a^	0.91 ^b^	14.57 ^b,c^	55.86 ^a,b^
Ostro	13.08 ^a^	5.19 ^a^	6.29 ^b^	515.16 ^a,b^	1.00 ^b^	15.11 ^c^	54.96 ^a^
Rouquin	13.84 ^a^	4.99 ^a^	5.57 ^a,b^	582.54 ^b^	0.69 ^a^	13.66 ^a,b^	55.19 ^a,b^
Schwabenkorn	12.58 ^a^	5.17 ^a^	5.89 ^a,b^	492.63 ^a,b^	0.87 ^a,b^	13.25 ^a^	56.66 ^b^
Oberkulmer-Rotkorn	14.00 ^a^	5.22 ^a^	6.67 ^b^	514.47 ^a,b^	0.88 ^b^	15.05 ^c^	54.64 ^a^
Average	13.25 ^A^	5.13 ^A^	5.77 ^A^	511.62 ^B^	0.87 ^A^	14.33 ^B^	55.45 ^A^
Growing season	<0.0001 ***	<0.0001 ***	0.2200 n.s.	<0.0001 ***	0.1230 n.s.	<0.0001 ***	<0.0001 ***
2018/2019	12.50 ^a^	4.92 ^a^	5.13 ^a^	542.48 ^b^	0.82 ^a^	15.25 ^b^	54.94 ^b^
2019/2020	14.52 ^b^	4.94 ^a^	6.42 ^b^	440.13 ^a^	0.91 ^a^	15.64 ^b^	53.26 ^a^
2020/2021	12.46 ^a^	5.74 ^b^	6.21 ^b^	596.49 ^b^	0.89 ^a^	10.98 ^a^	59.29 ^c^
Farming system	0.0110 *	<0.0001 ***	0.0430 *	0.0010 ***	0.3950 n.s.	0.0010 ***	0.0050 **
Hungary							
Conventional	12.74 ^a^	5.31 ^b^	6.17 ^b^	553.26 ^b^	0.84 ^a^	15.06 ^b^	53.94 ^a^
Organic	13.97 ^b^	5.31 ^b^	5.17 ^a^	525.04 ^a,b^	0.87 ^a^	13.08 ^a^	56.94 ^c^
Serbia							
Conventional	12.92 ^a,b^	4.60 ^a^	6.62 ^b^	439.92 ^a^	0.91 ^a^	15.10 ^b^	55.64 ^b^

***—*p* < 0.001, **—*p* < 0.01, *—*p* < 0.05, n.s.—not significant. Different uppercase letters within a column indicate significant differences (*p* < 0.05) between average values of spelt and bread wheat. Means followed by a common lowercase letter are not significantly different (*p* < 0.05) within each species.

## Data Availability

The datasets generated for this study are available on request to the corresponding author.

## Supplementary Materials

The following supporting information can be downloaded at: https://www.mdpi.com/article/10.3390/foods11244028/s1, Table S1: The average values for bioactive compounds, protein and starch content of bread and spelt wheat grown under conventional and organic farming system in Hungary and under conventional farming system in Serbia.
